# Fat–fat-free index in body mass assessment in young people

**DOI:** 10.3389/fphys.2022.947514

**Published:** 2022-08-24

**Authors:** Agnieszka Chwałczyńska, Aureliusz Kosendiak, Krzysztof Andrzej Sobiech, Waldemar Andrzejewski

**Affiliations:** ^1^ Faculty of Physiotherapy, Wroclaw University of Health and Sport Sciences, Wrocław, Poland; ^2^ Study of Physical Education and Sport, Wroclaw Medical University, Wrocław, Poland

**Keywords:** personalized fat–fat-free index, prevention of overweight, prevention of obesity, body composition, segmental body composition

## Abstract

The study aimed to personalize the classification of body weight using the fat–fat-free (FFF) index with the percentage of body fat and to develop classification standards for the FFF index for men aged 18–25 years. Moreover, 1,642 adolescents (1,200 ♀) were examined. Using body composition analyzers, weight was determined, as well as overall and segmental body composition. Based on the obtained values for fat mass and fat-free tissue mass, an overall FFF index was calculated. According to the BMI classification, 9% of ♀ and 6% of ♂ are underweight, 29% of ♀ and 13% of ♂ are overweight, and 5% of the subjects are obese. Women and men classified in the same group according to BMI differed statistically significantly in terms of body weight, FM%, and FFM. In contrast to BMI and FM%, the FFF used takes into account the ratio of fat mass to fat-free tissue and muscle tissue mass. The proposed classification of FFF was made taking into account the differences that arise with sexual development and physiological changes occurring in ontogeny. Assessment of body mass using the FFF index should be used as part of preventive screening for the early diagnosis and prevention of overweight and thus many chronic diseases for which overweight or obesity is a risk factor.

## Background

It has been suggested that excess body weight is a public health problem, affecting nearly two billion people over the age of 18 (Keating et al., 2014; Ng et al., 2014; Mahadevan and Ali, 2016; Duncan and Toledo, 2018; Yang et al., 2018; Kasman and Kasman, 2020). The incidence of overweight and obesity is such an economic burden on the health system, not only in terms of treating complications but also in terms of appropriately designed prevention, and should be individualized, taking into account the age, sex, possibilities (economic, housing, time, social, and physical), or health status of the patient, including predictive medicine, prevention of complications, and personalized medicine (PPPM/3P) (Golubnitschaja et al., 2021). However, the current system for the prevention of chronic diseases, the cause of which is undoubtedly excessive body weight, is only implemented at the primary health care (PHC) level within the framework of health balances for children at 2, 4, 6, 14, and 18 years of age. Thereafter, weight assessment in primary care is carried out incidentally in the case of hospital visits (the need for a specific dose of medication during treatment) or treatment of eating disorders. Often, the only control of body weight is its measurement at home or as part of personal training. It is on such measurements that weight reduction measures and thus the primary prevention of many chronic diseases, such as obesity, gastrointestinal cancers, degenerative joint changes, spinal overload, pain changes, or cardiovascular diseases, are often based. This is why it is so important to correctly diagnose weight abnormalities based on simple tools, where the focus of medical care is the patient and not the system. According to Polish statistics presented in reports by the Centre for Public Opinion Research (CBOS), almost 60% of citizens are overweight (Rutkowska, 2019). The same document also points out that in the younger group 12% of women and 5% of men are underweight, while in the older group, only 3% of women have a BMI < 18.5 kg/m^2^. Also, 71% of women and 61% of men aged 18–24 have normative body weight, while in the 25–34 age group, the percentages were 62 and 39%, respectively. According to the currently used BMI classification, the norms of the index do not change with age and are not gender-dependent. However, many authors point to the dependence of the weight–height ratio on age, for example, due to physiological changes in spinal curvature during ontogenetic or overload development (Guo et al., 1999; Bergman et al., 2011; Mialich et al., 2014; Agarwalla et al., 2015; Stupnicki, 2015; Malczyk, 2016; Chwałczyńska, 2017; Dwyer et al., 2020; Chwałczyńska and Andrzejewski, 2021). In standard studies by the WHO, CBOS, HBSC, COSI, or government institutions such as the Central Statistical Office, the percentage of overweight or obese people is mostly presented for children and adults, which introduces a kind of underestimation of the prevalence of body weight abnormalities. This is due to the use of a classification that does not take into account changes in age or gender-specific characteristics. To introduce prevention, measurements should be personalized and tools should be used that are adapted to the age and gender of the subject at the level of primary care or classification for competitive sport.

Increasingly, authors are emphasizing the role of body composition in the assessment of body mass abnormalities. Changes in fat mass requirements depend on age and sex and are related to fat mass function (Forbes, 1999; Guo et al., 1999; Hughes et al., 2001; Bergman et al., 2011; Kuen-Chang et al., 2011; Mialich et al., 2014; Gryko et al., 2019). As the BMI classification used to date does not take into account differences in sex, age, and body composition, researchers are attempting to use other methods based on anthropometric measurements (Sheldon typology and Stunkard figure rating scale), skinfolds (Szczawińska MT index), circumferential measurements, or using BAI or combined Mialich BMI fat methods (Heath and Carter, 1967; Slaughter et al., 1988; Bergman et al., 2011; Mialich et al., 2011; Burdukiewicz et al., 2015; Chwałczyńska, 2017; Lønnebotn et al., 2018; Gryko et al., 2019; Karpik et al., 2020; Vallieres et al., 2020; Chwałczyńska and Andrzejewski, 2021). However, all of these indices require a combination of at least two measurement methods, including peripheral skinfold measurements, which require considerable skill and precision of measurement, as well as equipment, and cannot be used in primary care because the primary care physician or nurse does not have enough time during the visit to take such measurements, and very often the skills. Waist circumference is often used to assess body weight normality as a method that is simple, easy to standardize, and clinically applicable. It is also recommended by the IAS and ICCR Working Group on Visceral Obesity as a complement to BMI in the assessment of abdominal obesity strongly associated with cardiometabolic mortality. However, its use is not widespread and is related to complementing a diagnosis based on BMI-based risk assessment. Waist circumference alone can indicate the presence of visceral obesity, the determination of which is important in many diseases. The IAS and ICCR working groups on visceral obesity also indicated that waist circumference should be measured according to the WHO protocol at the midthoracic level between the lower thoracic and iliac crest or according to the NIH protocol, that is, at the highest iliac crest. An expert panel whose work is presented in the recommendations of Ross et al. (2020) has developed preliminary values for waist circumference by gender and ethnicity. However, these are recommendations for physicians as a supplement to the diagnosis of morbid obesity. However, the problem with excessive body weight is not only abdominal obesity. An increase in fat mass at a constant body weight means a decrease in muscle mass and thus an increased problem with daily functioning due to reduced physical fitness. Not only being overweight or obese according to BMI but also a higher percentage of fat mass negatively affects cardiovascular fitness. (Chwałczyńska, 2017; Chwałczyńska and Andrzejewski, 2021).

The fat–fat-free (FFF) index proposed by Chwałczyńska takes into consideration the mass of adipose and fat-free mass, and the classification is presented in the form of a percentile grid, which allows for adjustment for age and gender (Chwałczyńska, 2017). This method can be used with any measuring tool that takes into account body composition components; it does not require special skills but only the introduction of one mathematical function to the electronic medical records of the patient, which will allow us, based on age, gender, height and weight, and the percentage of adipose tissue indicated on the simplest “bathroom weight”, to calculate the general FFF index with the determination of its regularity on the percentile grids for gender and age. The problem with the use of the FFF index is the lack of classification standards for men.

In the 21st century, one of the greatest challenges in healthcare is halting the epidemic of overweight and obesity, which not only affects healthcare costs but is also a risk factor for many diseases (Bhaskaran et al., 2018; Golubnitschaja et al., 2021). Reduction of treatment costs includes, among others, the implementation of overweight and obesity prophylaxis. For the prophylaxis to be targeted, it is necessary to estimate the body weight assessment taking into account gender, age, and body composition.

The study aimed to develop classification standards for the fat–fat-free (FFF) index for men aged 18–25. To achieve the main goal, the article presents a method of estimating the correctness of body mass based on its components, taking into account fat and lean mass. This method allows for individualization of the body mass classification based on the FFF index by adjusting the marginal values to the age and sex of the examined person.

The selection of the group at the age of 18–25 was dictated by the lack of contraindications to the tests and the absence of disease entities affecting the body composition or dependent on abnormal body composition. To generalize the results, people training in any sports discipline and people with noninfectious diseases such as type I diabetes, celiac disease, anorexia, bulimia, orthorexia, bigorexia, neoplastic diseases, and cardiovascular diseases were excluded from the study group.

## Materials and methods

The research was conducted in a university laboratory from 2015 to 2019, and 1,642 (women-1200 and men-442) people aged 18–25 were surveyed. The mean age of the subjects was 21.0 ± 2.1 years, mean height 169.8 ± 9.04 cm, mean body weight 64.4 ± 12.8 kg, and mean BMI 22.2 ± 3.4 kg/m^2^. To summarize the shortened BMI classification according to WHO (Gallagher et al., 2000; Bhaskaran et al., 2018), the subjects were divided into four groups: underweight group—UW (*n* = 119), group with normal body weight—NBW (*n* = 1,252), the third group—overweight—OW (*n* = 220), and group z obesity—OB (*n* = 51).

The criteria for inclusion in the study group were age 18–25 at the time of the study; no known pregnancy; no active neoplastic disease; no pacemaker; no bone anastomosis in the form of screws, wires, or plates; and no fungal skin changes in the area of feet and hands. The research was approved by the Bioethics Committee (No. 487/2006 and No. 12/2019).

**FIGURE 1:**
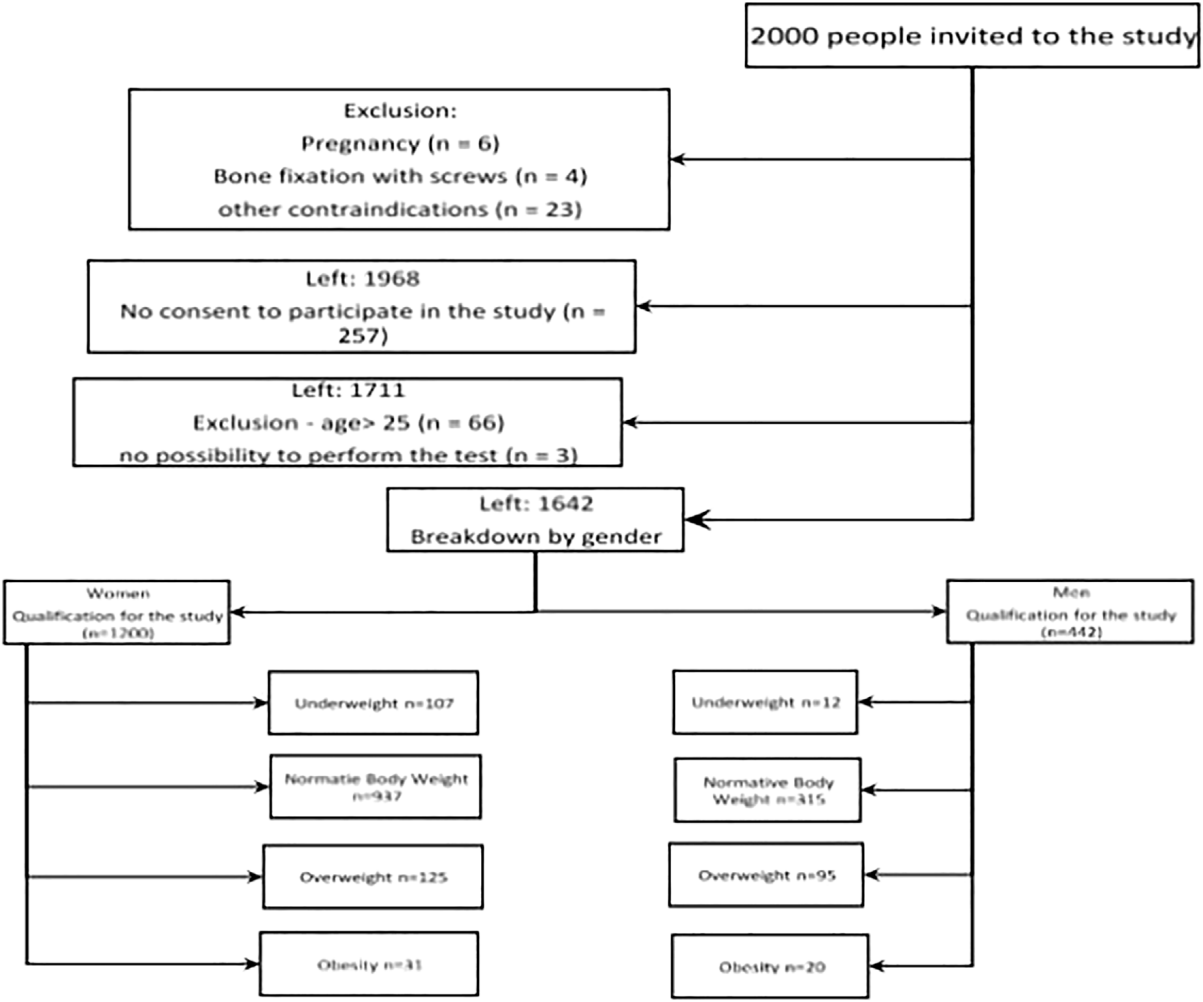
Flow diagram showing the process of qualifying for the study group.

The study was conducted by the assumptions of the Helsinki Declaration, and all respondents signed an informed, written consent to participate in the study with the use of electrical bioimpedance. Before the start of the study, they were informed about the course of the study and the possibility of stopping the measurement at any time, the need to refrain from high-proof alcohol or dehydrating drinks and intense physical activity at least 12 h before the test, and not eating (including drinks) at least 3 h before the test. The scheme of qualifying for the study is presented in the CONSOR scheme (Figure 1).

The body height was determined in the subjects with the SECA—213 height measuring devices with an accuracy of 0.1 cm. With the use of the Tanita 8-electrode body mass analyzer MC-780, weight and whole-body and segmental body composition were determined (fat mass percentage (FM%), fat mass (FM), and fat-free mass (FFM)). Weight and height index (BMI) and the general fat–fat-free index (FFF = FM/FFM) were calculated (Chwałczyńska, 2017).

**FIGURE 2:**
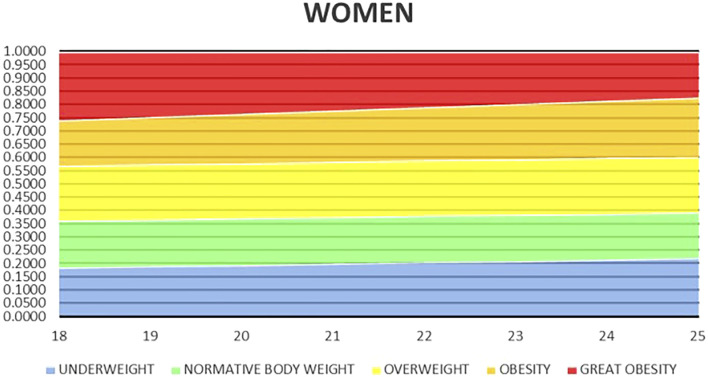
Percentile grid for general FFF for women aged 18–25 (Chwałczyńska, 2017).

Based on the results of the studies by Gallagher et al. (2000) and Sakamoto (2003), a classification for women and men, not including the Asian classification, was developed and used in the Gmon Health Monitor 3.4.2. software compatible with the Tanita body composition analyzer, determining the level of fat mass in four categories: low FM%, ♀ < 21% and ♂ < 8%; good FM%, ♀ 21–33% and ♂ 8–20%; increased FM%, ♀ 33–39% and ♂ 20–25%; and high FM%, ♀ > 39% and ♂ > 25%. Age and gender percentile grids were used to assess the overall FFF index in women (Figure 2) (Chwałczyńska, 2017).

Descriptive statistics were used to develop the research: mean value for a given quantity, standard deviation, and group size. The Shapiro–Wilk test showed that the distribution of the examined features was not normal. The groups were compared with the use of a nonparametric Mann–Whitney U test. The relationship between anthropometric data and the values of BMI, FFF, and FM% indices was investigated using the Spearman rank-order correlation. The level of statistical significance was set at *p* < 0.05.

## Results

Mean anthropometric values, such as height and weight, as well as BMI, of the studied women and men differed statistically significantly in individual groups according to the BMI classification in line with WHO guidelines (Table 1).

**TABLE 1:** Anthropometric data of the study group depending on gender and BMI classification.

			UW, *n* = 119		NBW, *n* = 1,252		OW, *n* = 220		OB, *n* = 51	
	♀ (*n* = 1,200)	♂ (*n* = 442)	♀ (*n* = 107)	♂ (*n* = 12)	♀ (*n* = 937)	♂ (*n* = 315)	♀ (*n* = 125)	♂ (*n* = 95)	♀ (*n* = 31)	♂ (*n* = 20)
Age (years)	21.1 ± 2.0	20.7 ± 2.62	20.7 ± 1.8	19.4 ± 0.7	21.1 ± 2.0	20.6 ± 2.1	21.0 ± 2.1	21.3 ± 2.2	21.2 ± 1.7	20.6 ± 2.5
P*	0.028		0.024		*p* < 0.001		0.334		0.243	
Height (cm)	165.9 ± 2.0	180.4 ± 7.0	165.2 ± 6.3	177.7 ± 8.3	166.2 ± 5.9	181.2 ± 7.0	164.2 ± 7.1	178.5 ± 6.6	167.6 ± 6.1	178.2 ± 5.9
P*	*p* < 0.001		*p* < 0.001		*p* < 0.001		*p* < 0.001		*p* < 0.001	
Weight (kg)	59.9 ± 10.0	76.7 ± 11.4	48.2 ± 4.6	56.3 ± 5.5	58.5 ± 6.3	73.2 ± 7.3	72.3 ± 7.0	85.0 ± 7.9	93.7 ± 11.4	106.2 ± 10.2
P*	*p* < 0.001		*p* < 0.001		*p* < 0.001		*p* < 0.001		*p* < 0.001	
BMI (kg/m^2^)	21.7 ± 3.2	23.6 ± 3.4	17.6 ± 0.7	17.8 ± 0.5	21.1 ± 1.7	22.3 ± 1.6	26.8 ± 1.4	26.6 ± 1.4	33.3 ± 3.1	33.5 ± 3.7
P*	*p* < 0.001		0.678		*p* < 0.001		0.584		0.961	

When comparing the values of FM% in the groups against the BMI classification, it was observed that there were statistically significant differences among men and women from one group. Among the studied men, only in the obese group did the average FM% value exceed the values indicated for a high amount of fat mass; in the remaining groups, the average value indicates a lower classification group. There were no statistically significant differences in the values of FM% and FFF in the group of men between the subjects underweight and NBW. FM% and FFF values did not differ statistically significantly in the groups of OW and OB women. There were statistically significant differences in the remaining groups (Table 2).

**TABLE 2:** Comparison of the mean percentage of the amount of body fat (FatP) and the FFF index in relation to gender and BMI.

	FatP			FFF		
	♀	♂	♀ Vs. ♂	♀	♂	♀ Vs. ♂
UW, n = 119	17.1 ± 4.2	6.3 ± 2.5	*p* < 0.001	0.208 ± 0.059	0.068 ± 0.028	0.118
NBW, n = 1,252	24.5 ± 4.9	13.3 ± 4.1	*p* < 0.001	0.330 ± 0.087	0.156 ± 0.055	*p* < 0.001
OW, n = 220	34.0 ± 4.3	19.2 ± 3.9	*p* < 0.001	0.523 ± 0.098	0.240 ± 0.06	*p* < 0.001
OB, n = 51	41.7 ± 5.1	27.6 ± 4.9	*p* < 0.001	0.729 ± 0.151	0.387 ± 0.085	0.070
UW vs. NBW	*p* < 0.001	NS		*p* < 0.001	NS	
UW vs. OW	*p* < 0.001	*p* < 0.001		*p* < 0.001	*p* < 0.001	
UW vs. OB	*p* < 0.001	*p* < 0.001		*p* < 0.001	*p* < 0.001	
NBW vs. OW	*p* < 0.001	*p* < 0.001		*p* < 0.001	*p* < 0.001	
NBW vs. OB	*p* < 0.001	*p* < 0.001		*p* < 0.001	*p* < 0.001	
OW vs. OB	NS	*p* < 0.001		NS	*p* < 0.001	

Having applied the classification of body mass abnormalities according to BMI in line with WHO guidelines, it was stated that the study group comprised 8.9% of women and 2.7% of men who were UW, 78.1% of women and 71.2% of men with NBW, 10.4% of women and 21.5% of OW men, and 2.6% of women and 4.5% of men with OB. Using the classification according to the percentage of fat mass, low FM% was found in 26% of women and 9.3% of men, good FM% in 62% of women and 73.3% of men, and increased FM% in 8.8 and 12.7%, respectively, and high FM% in 3.2 and 4.75% of the subjects.

**FIGURE 3:**
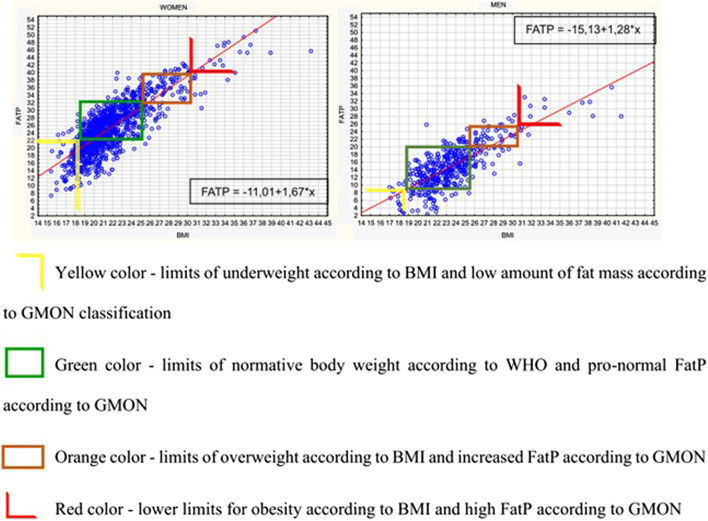
Scatterplot with trendline and regression equation for FatP versus BMI by gender.

Juxtaposed classification of BMI according to WHO and the FM% indicated slight discrepancies in the size of the groups in particular classification ranges. The largest differences were observed among women, where less than 9% of the respondents were underweight according to the WHO classification, and assuming the classification according to fat mass, as many as 26% of the respondents had FM% close to age and gender. Similarly, in the group of men, three times more subjects had low-fat mass values (less than 8%) than men, whose BMI was lower than 18.6 kg/m^2^, that is, underweight according to the BMI classification. Using the scatter plot of FM% values depending on the BMI classification, it can be observed that the trend line takes an upward position both in the group of women and men. When superimposing the classification range for body weight abnormalities by BMI and the age- and sex-adjusted classification for FM% on the graph, a significant group of women was observed in which the classifications did not coincide (Figure 3).

After superimposing the FM% classification on the BMI classification, it was found that 85% of women and 66.7% of UW men also had a reduced value of body fat. In the group of people with an NBW according to BMI, 72.6% of women and 85.1% of men had the correct amount of fat mass, adequate for the NBW. The highest percentage of non-corresponding classifications was observed in the group of OW people: 49.4% of women and 62.1% of men—most frequently the subjects in this group—had the correct amount of fat mass (37.6 and 54.7%, respectively) (Table 3).

**TABLE 3:** Comparison of the classification of body mass abnormalities according to BMI with the classification according to BMI combined with FatP depending on gender.

		♀ [%]				♂ [%]			
		BMI	BMI + FatP**	BMI + FatP*	FFF	FFF	BM + FatP*	BMI + FatP**	BMI
*UW*	UW+ low FatP	*8.92*	85.0	7.6	8.75	6.1	1.8	66.7	*2.71*
	UW + good Fatp		15.0					33.3	
	UW + increased FatP		0.0					0.0	
	UW + high FatP		0.0					0.0	
*NBW*	NBW+ low FatP	*78.08*	23.6		56.9	76.2		9.8	*71.27*
	NBW + good FatP		72.6	56.7			60.9	85.1	
	NBW + increased FatP		3.7					4.8	
	NBW + high FatP		0.1					0.3	
*OW*	OW+ low FatP	*10.42*			29.25	12.7		2.1	*21.49*
	*OW* + good FatP		37.6					54.7	
	*OW*+ increased FatP		50.4	5.3			8.1	37.9	
	*OW*+ high FatP		12.0					5.3	
*OB*	OB+ low FatP	*2.58*	0.0		5.1	5.0		0.0	*4.53*
	*OB* + good FatP		3.2					5.0	
	*OB*+ increased FatP		25.8					20.0	
	*OB*+ high FatP		71.0	1.8			3.4	75.0	
*[%]*		100		71.4	100	100	74.2		100

(*taking into account all subjects of a given gender and **taking into account the subjects classified to a given group of body mass abnormalities according to the BMI classification).

Values in italics are consistent with the classification adopted and used so far—they can be harmonized with other values in the table.

The research employed the classification of the FFF index for women. Compared to the classification, taking into account the combination of the BMI and the FM%, which excluded 30% of women and 25% of men from the classification, it can be observed that FFF, like BMI, includes all of the subjects (Table 3).

In women, a statistically significant weak correlation was observed between age and FM% (*p* = 0.083) and the FFF index (*p* = 0.079). In the case of men, a statistically significant weak correlation was noted between age and BMI. The correlation between BMI and FM% (♀0.779 and ♂0.719), BMI and FFF (♀0.779 and ♂ 0.719), and FM% and FFF (♀0.997 and ♂ 0.999) is statistically significant and very high in both sexes.

The examined women classified as being UW, NBW, and OW, according to the FFF classification, had statistically significantly lower BMI values compared to men. On the other hand, men in all groups according to the FFF classification had statistically significantly lower values of FM% than women. When comparing the values of FM% and BMI in individual groups according to the FFF classification, statistically significant differences were found between all groups among women and men, and this significance was not present only in OW and OB subjects (Table 4).

**TABLE 4:** Comparison of FatP and BMI values depending on the FFF classification in the studied women and men.

	BMI					FatP		
	*n*	♀	*n*	♂	♀ Vs. ♂	♀	♂	♀ Vs. ♂
UW, *n* = 132	105	18.7 ± 1.4	27	20.1 ± 1.7	*p* < 0.001	14.1 ± 2.1	4.9 ± 2.1	*p* < 0.001
NBW, *n* = 1,020	683	20.5 ± 1.6	337	22.8 ± 2.2	*p* < 0.001	22.6 ± 2.9	13.8 ± 3.3	*p* < 0.001
OW, *n* = 407	351	23.7 ± 2.3	56	26.5 ± 2.2	*p* < 0.001	30.9 ± 2.6	21.9 ± 1.4	*p* < 0.001
OB, *n* = 83	61	30.9 ± 4.0	22	32.1 ± 4.9	0.058	40.9 ± 3.4	28.4 ± 2.9	*p* < 0.001
UW vs. NBW		*p* < 0.001		*p* < 0.001		*p* < 0.001	*p* < 0.001	
UW vs. OW		*p* < 0.001		*p* < 0.001		*p* < 0.001	*p* < 0.001	
UW vs. OB		*p* < 0.001		*p* < 0.001		*p* < 0.001	*p* < 0.001	
NBW vs. OW		*p* < 0.001		*p* < 0.001		*p* < 0.001	*p* < 0.001	
NBW vs. OB		*p* < 0.001		*p* < 0.001		*p* < 0.001	*p* < 0.001	
OW vs. OB		*p* < 0.001		*p* < 0.001		0.506	0.506	

## Discussion

In today’s world, the concept of healthy body weight is on everyone’s lips. Nutritionists stress that the most important thing for maintaining health and well-being is a proper diet. Personal trainers and physical culture representatives emphasize the importance of exercise. Both are partly right. Determining individual energy requirements should be part of predictive and preventive medicine as part of 3P medicine (Golubnitschaja et al., 2021). Maintaining a healthy body weight requires both a balanced diet and physical activity. However, all dietitians, physicians, physiotherapists, and trainers must rely on normative values to identify any abnormalities. By adopting the BMI classification used to date, we are overlooking very important aspects of the physiological changes taking place in the human body. Research shows that the weight–height index cannot be used without taking into account gender and age differences. The significant reduction in the number of people with normative body weight over 10 years, at the age of human activity, shows that the use of norms is wrong. Chwalczynska’s work showed that, with unchanged body weight during ontogenetic development, women change their BMI value due to a decrease in body height; they did not gain weight but decreased height, which automatically increased BMI (Chwałczyńska, 2017). Similar results were obtained by researchers determining the health and well-being of elderly people, their lack of disease, and well-being with a body weight–height ratio of 24–29 kg/m2, that is, according to the classification, their body weight indicated being OW (Guo et al., 1999; Agarwalla et al., 2015; Chwałczyńska, 2017; Dwyer et al., 2020).

A comparison of the mean amount of fat mass for men and women classified into one group according to BMI shows statistically significant differences in all groups. The use of a single BMI classification results in the lack of an individualized approach to the problem of abnormal body weight and thus the possibility of underestimating the problem of hidden overweight, which should be monitored in terms of primary health care and prevention of diseases whose risk factor is excessive fat mass. Many authors point to the need to differentiate BMI by age and gender. The higher percentage of fat mass in women compared to men is physiological, due to a different anatomical structure and fat mass requirements. However, BMI does not take this into account, classifying both sexes according to a single weight-to-height ratio. The problem relates not only to the gender and age divisions but also to the standardization of BMI values worldwide without taking into account the ethnic variation in physique caused by the climatic conditions in which the subjects live (different physiques of Central Africans, East Asians, or Northern Europeans). This is why an increasing number of authors are questioning the WHO classification of body mass abnormalities, especially in seniors or in gender comparisons (World Health Organization Obesity, 2000; Deurenberg et al., 2002; World Health Organization Expert Consultation, 2004; Gupta and Kapoor, 2012; Bhogal and Langford, 2014; Chwałczyńska, 2017; Silva et al., 2017; Christensen et al., 2018; Mialich et al., 2018; Provencher et al., 2018; Chwałczyńska and Andrzejewski, 2021; Zhu et al., 2022).

The inadequacy of BMI as a classification tool can also be observed by comparing FM% values for women of different ages, as presented in the study by Chwałczyńska (2017). Estimating body mass abnormalities using FM% distorts the whole-body picture. Using only the percentage of fat mass in total body weight to assess abnormalities, the aspect of too much muscle mass is overlooked. For example, people involved in strength sports or bodybuilding have a fat mass in the lower limits of the norm (♂ 8% FM or ♀ 15% FM), but their body mass is more than 92%, with 85% excessively composed of muscle mass. This ratio of FM to MM is dangerous for health, including cardiovascular health. A similar FM score to that of a bodybuilder can be had by a nontraining man with a light build, or a long-distance runner or swimmer, but their MM will be much lower. It is therefore important to use the ratio of FM and FFM to assess the correctness of body mass. Only the combination of these two values, that is, total body mass and body composition, can give a complete picture of normal body mass. Attention to the need to assess the ratio of MM to FM becomes important for adolescents undertaking individual physical activity to reduce weight or maintain health and well-being. Many authors have observed a decrease in the number of overweight and obese women aged 15–25 years and an increase in the number of underweight women (Dietz, 2004; Lazzeri et al., 2008; Chwałczyńska, 2017; Dereń et al., 2018; Wang et al., 2021). Fashion and lack of control by primary health care are important contributors to this situation. Women deprived of medical control self-impose diets that are very often not adapted to their needs and judge themselves only based on BMI without attempting to assess their bodies. Even in the study group, which was recruited from medical students with knowledge of physiology and the role of fat mass in the human body, a group of women with a fat mass of less than 10% of total body weight was observed. This may indicate restrictive diets and/or a lack of control over the correct choice of physical activity or its very high intensity. Among the subjects, a rather large group of people were also observed, not only women but also men, with a high FM% and BMI indicating underweight or lower NBM. By drawing attention to the need to control not only body weight or FM% but also the ratio of FM to FFM, we are carrying out primary prevention of further disorders resulting from too high MM or too low FM. It should be mentioned here that the cessation of intensive exercise without the supervision of competent people—personal trainers and sports doctors—can lead to a rapid loss of muscle mass being replaced by fat mass and, consequently, the onset of obesity. Therefore, monitoring the FM-to-FFM ratio, that is, assessing the FFF index, is important not only for the prevention of overweight and obesity in working people but also for athletes at the end of their careers.

Observing the dispersion of fat mass values according to BMI, there is a large group of women whose BMI indicates a normative body weight and whose fat mass is reduced. In this group, the mean FM% is 18.0 ± 2.4, while in the group classified as underweight based on BMI with normal FM%, the mean is 23.6 ± 2.2. Reduced fat mass in the study group of women is not only a matter of nomenclature but also a health problem. Too low FM, such as eating disorders in young women, negatively affects their reproductive potential, the course of pregnancy, and the health of the child after birth. The negative effects of too low FM also include osteopenia, vitamin deficiencies, and general irritability. Reduced fat mass in the study group affects the proper functioning of women during the reproductive period, which can lead to infertility or miscarriage (Chwałczyńska, 2017; Makowska-Donajska and Hirnle, 2017; O'Brien et al., 2017; Tabler et al., 2018; Kimmel et al., 2016; Chwałczyńska et al., 2018).

The problem of infertility affects 30 percent of women and 25 percent of men, indicating the need for the PCP to include these individuals in the biomonitoring of the 3P drug. Such significant numbers indicate the need to combine indicators that take into account not only weight and height values but also body composition. One such tool is the FFF. Authors relying on this indicator in their studies have demonstrated its versatility not only for one-off ongoing assessment but also for assessing changes in the training or ontogenetic process (Milewska et al., 2016; Chwałczyńska, 2017; Chwałczyńska et al., 2018; Rutkowski et al., 2020; Chwałczyńska and Andrzejewski, 2021; Sojka et al., 2022). FFF is age- and sex-dependent and takes into account MM, which is part of lean mass. The similar MT index was based on skinfold measurements, which limits its applicability to people skilled in the use of body fat calipers. For the Mialich index based on a combination of BMI and muscle mass, there is a problem with the reliability of MM measurement. Bioimpedance devices equally pick up smooth and striated muscle and indicate predicted muscle mass, which is most often calculated by subtracting fat-free dry bone mass. The criterion of body mass abnormality proposed for this index is not corrected for either age or sex (Slaughter et al., 1988; Mialich et al., 2011). The MT or Mialich indices only partially implement the principle of 3P medicine, their main drawbacks being the lack of universal access to measurement tools and the lack of adaptation to the individual subject in terms of gender or age, which is characteristic of the FFF index.

The index recommended by the IAS and ICCR Working Group on Visceral Obesity is the waist circumference, but this index does not take into account the distribution of fat mass and only indicates the presence of abdominal obesity. Its use is therefore limited to assessing the risk of cardiovascular abnormalities and not, as in the case of the FFF index, to assessing the distribution and possible changes in the individual components of body composition. The BMI index is very generalized and does not take into account age and gender differences, the MT index is based on skin-fat fold measurements, which requires anthropometric knowledge, the Mialich index is based on predicted muscle mass, which is inaccurate, and the peripheral index is only indicated for assessing abdominal obesity. The use of the FFF index allows it to be adjusted for age, gender, and changes occurring not only in the overall physique but also in individual body segments. It is simple and noninvasive and can be used in any body composition assessment tool.

At the same time, it is worth noting the lack of statistically significant differences between FFF values for underweight men and NBW according to the BMI classification (Table 2), which may indicate reduced body weight in underweight men with a normal internal body structure. Such information is very important for those developing dietary regimens, as in cases of being underweight, the diet should include caloric supplementation. Nevertheless, the FFF index for women with OW is not statistically significantly different from the obese group (Table 2), which may indicate that fat mass does not change significantly in these groups and that obese women primarily gain total body weight in both adipose and lean tissue. For the FFF classification used, statistically, significant differences were observed between BMI values in all study groups (Table 3). This confirms the assumption that this index differentiates body mass abnormalities more clearly than BMI, taking into account body composition components. When comparing the percentage fat mass in the FFF classification groups, it was found that the differences were not significant in the OW and obese groups (Table 4). This supports the hypothesis that in the OW and obese groups, the subjects have a similar percentage of fat mass and that the excess weight is equally due to fat mass and fat-free mass. A small percentage difference in high body mass results in a significant difference in fat mass. For a man 180 cm tall and weighing 100 kg, 30% FM% equates to 30 kg of fat mass and FFF = 0.428, while a change of 1%—consequently to 31 kg of body fat—increases the FFF to 0.449. The same index values are obtained when the body weight is 75 kg and FM% remains at 30%/31%. In the first case, the difference in FM weight is 1 kg, and in the second case it is 0.75 kg; therefore, the heavier man is classified by BMI as OB (BMI = 30.86) and the other as NBW (BMI = 23.1), but the problem remains the same—high FM%. Using the FFF index, the ratio of fat mass to lean mass remains decisive, which in both cases indicates OW in men in this age group. For 3P medicine, it is important to approach the problem from an individual aspect rather than a general one, and therefore, the proposed FFF index may serve to better identify individuals who qualify for the problem of weight abnormalities than the BMI index used to date. At the same time, the introduction of the FFF index into primary care does not require a large financial investment and will allow for more precise identification of the problem of excessive body weight.

**FIGURE 4:**
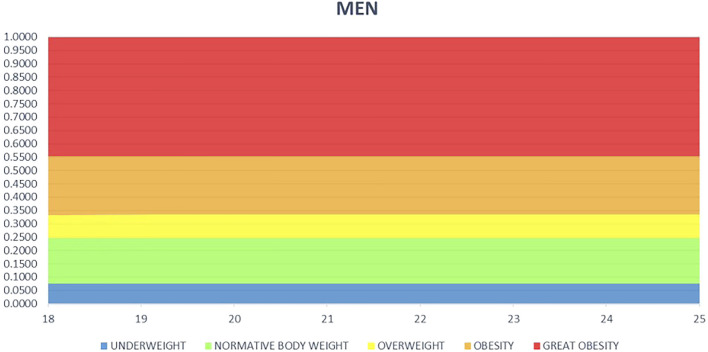
Percentile grid for general FFF for men aged 18–25.

The research presented here was aimed at developing preliminary classification values of the FFF index for men aged 18–25 years. The development of percentile grids of the FFF index for men aged 18–25 years will allow for the implementation of prevention aimed at the problem of body weight abnormalities not only among researchers but also in health centers or as part of training in gyms and sports halls under the guidance of qualified trainers. Based on the study, it was proven that the FFF index for men is statistically significantly lower in the OW and normal weight groups than for women. No statistically significant gender differences were observed in the underweight and obese groups. The differences obtained confirm the need to distinguish the gender classification of body weight. Lower FFF indices indicate higher fat-free mass. In developing the percentile grid for the FFF index, as with the percentile grid for women, the Steinhaus algorithm was used, supplementing the BMI classification with further values (Figure 4).

The proposed classification is limited to 18–25 years, as this is the only age group of the men surveyed; therefore, the research project requires further research to complement it with other age groups.

The FFF index can be a good tool to assess the changes that occur in a man’s body without a change in body weight. The FFF index is also a good tool to assess the changes that occur in physioprevention programs for overweight and obesity in children implementing 3P medicine, targeting the individual young patient. Using the FFF index, positive changes were observed in children under the influence of training, which were not visualized by a change in body weight. Similarly, in the authors’ study of a group of students assessing the changes that occurred under the restrictions imposed by the Covid-19 pandemic, it was observed that these changes occurred in body composition components and not in body weight. In assessing these changes, it was found that the percentage of fat mass increased at the expense of lean mass (Chwałczyńska et al., 2017; Rutkowski et al., 2019; Chwałczyńska and Andrzejewski, 2021; Golubnitschaja et al., 2021; Sojka et al., 2022).

## Conclusion

The BMI index is not age- and sex-adjusted and therefore does not take into account the regularities of ontogenetic changes and sexual dimorphism in the structure of human body tissues. This method, which is currently used for the prevention of overweight and obesity, is imperfect and excludes from intervention people who meet the weight standards and whose abnormalities relate to a lack of proper body composition proportions.

The classification based on FM% does not take into account total body weight but only shows the percentage of fat mass, which is not adequate when it comes to the problem of excessive body weight. This is evidenced by the lack of statistically significant differences between the OW and obesity groups for women and the underweight and NBW groups for men.

The FFF index used was based on the ratio of fat mass and lean mass, also taking into account muscle tissue mass. The proposed FFF classification was developed taking into account the differences that arise with sexual development and the physiological changes that occur during ontogeny.

The FFF index can be used as part of PPPM medicine because it is simple and individualized, its use does not require specialized equipment, it is safe, and can be used prophylactically, taking into account individual variability and lifestyle.

### Expert recommendations

Body mass assessment using the FFF index should be used as part of preventive examinations carried out in occupational medicine as periodic examinations for every employee to identify and prevent overweight at an early stage and thus prevent many chronic diseases whose risk factor is overweight or obesity. Assessment of the FFF index will allow for changes in the body composition of the subject to be observed and early detection of latent overweight, characterized by an increase in fat mass with stabilized total body weight. Prompt diagnosis will not only change the possibility of overweight or obesity-related complications but will also reduce the financial cost of subsequent medical care, as it is cheaper and more important to act preventively than to treat obesity—prevention is better than cure.

The FFF index should also be used to assess body composition in competitive sports, especially segmental indices. The use of FFF will allow for the assessment of symmetry in the distribution of body mass components.

## Data Availability

The original contributions presented in the study are included in the article/Supplementary Materials; further inquiries can be directed to the corresponding author.
